# Trimethoprim‐Sulfamethoxazole in the Treatment of Idiopathic Granulomatous Mastitis: A Retrospective Cohort Study

**DOI:** 10.1002/hsr2.73024

**Published:** 2026-08-10

**Authors:** Soheila Sayad, Saba Ahmadi, Mahdi Ahmadinia, Azadeh Eshraghi, Hamidreza Aslani, Maryam Farasatinasab

**Affiliations:** ^1^ Department of Surgery, School of Medicine Iran University of Medical Sciences Tehran Iran; ^2^ Firoozgar Clinical Research Development Center (FCRDC) Iran University of Medical Sciences Tehran Iran; ^3^ Department of Clinical Pharmacy and Pharmacoeconomics, School of Pharmacy Iran University of Medical Sciences Tehran Iran; ^4^ Lung Transplantation Research Center, National Research Institute of Tuberculosis and Lung Diseases (NRITLD) Shahid Beheshti University of Medical Sciences Tehran Iran

**Keywords:** antibiotic therapy, corticosteroids, idiopathic granulomatous mastitis, steroid‐sparing strategy, trimethoprim‐sulfamethoxazole

## Abstract

**Background and Aims:**

Idiopathic Granulomatous Mastitis (IGM) is a rare, chronic inflammatory breast condition with uncertain etiology and challenging management. Standard treatments often involve corticosteroids or surgery, both associated with significant relapse and adverse effects. Recent interest has emerged in using antibiotics such as Trimethoprim‐Sulfamethoxazole (TMP‐SMX) due to potential antimicrobial and anti‐inflammatory benefits, though evidence remains limited.

**Methods:**

This retrospective cohort study evaluated the effectiveness and safety of TMP‐SMX compared to conventional therapies in female patients diagnosed with histologically confirmed IGM. Patients were categorized into the TMP‐SMX and control groups. Primary outcomes included treatment response and recurrence rates; secondary outcomes assessed changes in lesion size, clinical severity, and adverse events. Statistical analyzes included Chi‐square, Fisher's exact test, and odds ratios (ORs) with 95% confidence intervals.

**Results:**

Complete clinical remission was achieved in 70.8% of TMP‐SMX (*N* = 24) patients versus 51.2% in the control (*N* = 43) group (*p* = 0.12). Recurrence rates were comparable between groups (37.5% in the TMP‐SMX group vs. 27.9% in the control group, *p* = 0.51). TMP‐SMX patients showed trends toward reduced lesion size and milder disease, although differences were not statistically significant. Adverse events were less frequent in the TMP‐SMX group, except for transient fatigue (*p* = 0.04).

**Conclusion:**

This study provides evidence that TMP‐SMX is a safe and clinically effective alternative to corticosteroid‐based regimens for managing idiopathic IGM. TMP‐SMX demonstrated comparable efficacy, including favorable trends in lesion regression and disease severity improvement, with notably fewer systemic adverse events.

**Abbreviations**
CI: confidence interval
DMARDs: disease‐modifying antirheumatic drugs
IGM: idiopathic granulomatous mastitis
NSAIDs: non‐steroidal anti‐inflammatory drugs
OR: odds ratio
SPSS: statistical package for the social sciences
TMP‐SMX: trimethoprim‐sulfamethoxazole

## 1 Introduction

IGM is a rare, benign inflammatory breast disease characterized by chronic granulomatous inflammation with sterile abscess formation. It typically affects young women of childbearing age, often within a few years postpartum, and commonly presents as a unilateral breast mass with pain, erythema, and occasionally sinus tract formation [1]. Clinically, IGM can closely mimic infectious mastitis or even breast cancer, making diagnosis [1, 2]. The diagnosis is confirmed by histopathology showing non‐caseating granulomas after excluding known infectious causes [1, 2, 3]. Despite five decades of reported cases, the etiopathogenesis of IGM remains unclear. An exaggerated autoimmune reaction is a leading hypothesis [1, 4], but the disease's “idiopathic” nature suggests no definitive trigger; even so, some studies have implicated *Corynebacterium spp*. as a potential instigating factor in a subset of patients [5]. This uncertainty in etiology underlies the ongoing debate in management strategies.

Various therapeutic strategies have been utilized in the management of IGM; however, each approach presents notable limitations, and a universally accepted treatment protocol has yet to be determined [1]. Corticosteroids represent the most frequently utilized initial treatment option and have the potential to achieve remission in a significant proportion of patients [6]. Nonetheless, the administration of steroid therapy frequently requires an extended duration and is linked to recurrent relapses as well as significant systemic side effects [1, 6, 7]. Immunosuppressants like methotrexate are utilized in challenging cases or to minimize steroid use, with evidence indicating significant response rates over extended treatment periods [8, 9]. Although promising, immunosuppressive therapy requires months of administration and carries risks. Surgical intervention, ranging from abscess drainage to wide excision or even mastectomy, has also been used for localized or severe disease. Surgery can achieve definitive control in some cases, but recurrence rates post‐excision range widely (0%–50%) and surgery may result in scarring and other morbidities [1, 10]. Since IGM may resolve on its own with time, observation alone is occasionally tried in mild cases; however, this approach carries the risk of prolonged symptoms and is not always appropriate. Treatment decisions are frequently made based on clinical judgment and experience in the absence of high‐quality evidence or guidelines because all of these modalities have significant disadvantages and varying degrees of success, making IGM management difficult [11].

In recent years, antibiotic therapy, especially with Trimethoprim‐Sulfamethoxazole (TMP‐SMX), has gained attention as a possible alternative or complementary treatment for IGM. This interest stems from the similarities between IGM and bacterial infections, particularly the identification of organisms such as *Corynebacterium kroppenstedtii* in certain lesions [5]. A case series involving 20 patients demonstrated clinical improvement in the majority of cases, with only 30% experiencing relapse within a 6‐month period. This finding indicates a more favorable recurrence profile when compared to steroid treatments [12]. This suggests that while TMP‐SMX may offer advantages for certain patients, its overall effectiveness as a standalone treatment is constrained. Consequently, the evidence regarding TMP‐SMX in IGM presents a varied picture, with studies yielding inconsistent results.

Regarding the use of TMP‐SMX for IGM, there are still a number of significant information gaps. In the first place, it is unclear how TMP‐SMX could help with IGM. Since an autoimmune etiology is suspected in the majority of IGM patients and no obvious infectious agent is found, it is uncertain whether TMP‐SMX is working by eliminating an occult microbe or by having an anti‐inflammatory impact [3, 5, 10], Second, there aren't enough solid research to support TMP‐SMX; the majority of published data for it comes from case reports or series that don't include control groups, and no randomized trials have been conducted to determine its effectiveness in comparison to traditional therapies [5]. Additionally, doctors are unable to identify which patients are more likely to respond to antibiotics than immunosuppressive medication or surgery since there are currently no recognized predictors of therapeutic response in IGM. These ambiguities make clinical judgment more difficult and emphasize the need for more evidence‐based recommendations.

This study seeks to assess how effective TMP‐SMX is in treating IGM in comparison to standard management practices. A retrospective cohort study was conducted to examine the treatment outcomes of patients with IGM who received TMP‐SMX in comparison to other treatment options. The study seeks to elucidate the therapeutic value of TMP‐SMX by examining the clinical remission and relapse rates among the groups. This investigation aims to address current knowledge gaps and ultimately provide data that could guide future treatment strategies for idiopathic granulomatous mastitis.

## 2 Materials and Methods

### 2.1 Study Design and Setting

This retrospective cohort comparative study took place at the Breast Surgery Outpatient Clinic of Firoozgar Hospital, which is associated with Iran University of Medical Sciences in Tehran, Iran. The research spanned a period of 5 years, from 2018 to 2023, during which the medical records of patients diagnosed with IGM were meticulously examined.

The main aim of this study was to evaluate the clinical outcomes of patients receiving treatment with TMP‐SMX in comparison to those undergoing standard therapies, which include corticosteroids, Non‐Steroidal Anti‐Inflammatory Drugs (NSAIDs), immunosuppressants, or surgical interventions. The confirmation of IGM diagnosis was achieved through histopathological reports and a thorough clinical evaluation conducted by breast surgeons. We carefully reviewed all accessible patient files, pathology results, imaging studies, and physician follow‐up notes to gather pertinent information.

The research team utilized a structured checklist for data collection, which effectively captured essential variables including patient demographics, disease severity, treatment type and duration, therapeutic response, recurrence, and complications. Trained clinical staff manually extracted data to guarantee both completeness and accuracy.

This study adhered to the ethical principles of the Declaration of Helsinki and was approved by the institutional ethics committee of Iran University of Medical Sciences (Ethical Number: IR.IUMS.REC.1403.1012). Given the retrospective design of the study, the requirement for written informed consent was waived by the Ethics Committee. Patient confidentiality was strictly preserved by anonymizing all data before analysis. The minimum sample size was determined based on the prevalence of IGM cases referred to the clinic during the study period and the feasibility of complete case retrieval from archived records.

### 2.2 Study Population

**Figure 1:**
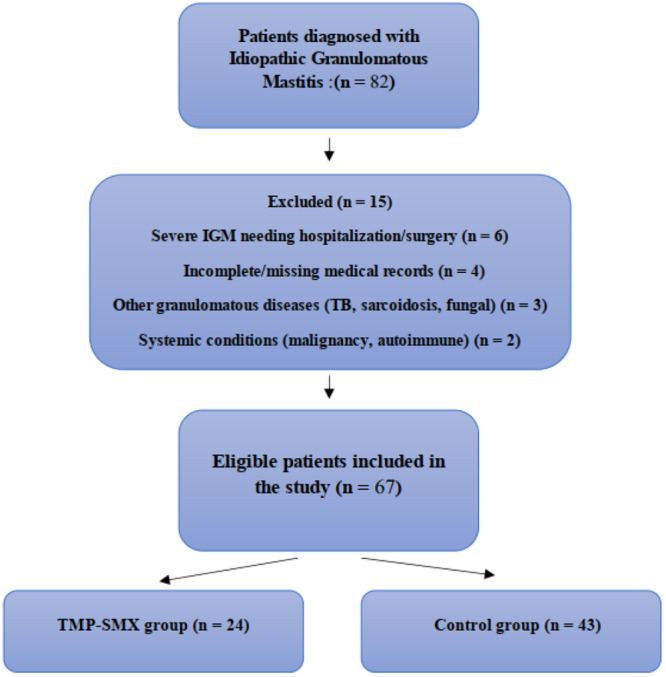
Flow diagram of patient enrollment.

Between 2018 and 2023, a total of 82 female patients with a diagnosis of idiopathic granulomatous mastitis (IGM) were initially identified. Following application of the predefined inclusion and exclusion criteria, 67 patients were deemed eligible and included in the study. Of these, 24 were assigned to the TMP‐SMX group and 43 to the control group. The detailed patient selection process is illustrated in Figure 1.

### 2.3 Treatment Groups and Interventions

Patients were classified into two groups according to their primary treatment modality. The TMP‐SMX group received oral trimethoprim‐sulfamethoxazole (80/400 mg twice daily) for a minimum of 4 to 6 weeks. Treatment duration was tailored based on clinical response, which was regularly assessed by a breast surgeon. The control group included patients who received standard treatments, which encompassed systemic corticosteroids (for instance, prednisolone at a dosage of 30–40 mg per day, tapered over 6–12 weeks), methotrexate (administered at 7.5–15 mg per week alongside folic acid), nonsteroidal anti‐inflammatory drugs (NSAIDs) for symptom relief, or surgical procedures like drainage or excision. Treatment decisions were tailored to each patient, taking into account the severity of the disease and their previous responses to therapy. Individuals who received treatment exclusively with NSAIDs and did not undergo any active antimicrobial or immunosuppressive therapy were not included in the analysis. The outcomes of treatment, such as remission, recurrence, and adverse effects, were then compared between the two groups.

### 2.4 Data Collection and Outcome Measures

The research team extracted patient data from archived medical records, which encompassed clinical charts, pathology reports, and imaging files, utilizing a standardized checklist for this purpose. The checklist encompassed various aspects such as demographic characteristics, the severity of the disease, the type and duration of treatment, therapeutic responses, and any complications that were documented. For patients receiving TMP‑SMX, routine laboratory monitoring was also documented as part of the data collection process. Baseline tests included complete blood count and renal and hepatic function panels, followed by repeat assessments at scheduled follow‑up visits during therapy. These tests were performed to identify potential drug‑related adverse effects, such as cytopenias or elevations in creatinine or liver enzymes. Inclusion criteria were strictly applied, permitting only those patients who possessed comprehensive documentation and had undergone a minimum follow‑up period of 1 year. For each patient, the exact duration of follow‑up was recorded to facilitate an accurate assessment of remission and recurrence outcomes. The collection of data was carried out by trained clinical personnel, aimed at ensuring both consistency and accuracy throughout the records. The severity of the disease was determined through clinical assessments and ultrasonographic evaluations. Mild IGM presented with lesions that were less than 2 cm in size, exhibiting neither fistula formation nor ulceration. In contrast, moderate cases were defined by lesions ranging from 2 to 5 cm, accompanied by a solitary fistula and minimal discharge.

The therapeutic response was assessed through clinical improvement and classified into four predetermined outcomes. Clinical outcomes were assessed during routinely scheduled follow‑up visits (typically every 4–8 weeks), and all response categories were applied within this standardized assessment schedule. Complete and partial responses were determined based on clinical examinations conducted during these visits, whereas relapse was defined as the reappearance of symptoms after a documented complete response at any point during the mandated ≥ 6‑month follow‑up period. A complete response is characterized by the total resolution of clinical signs and symptoms, signifying a full remission of the disease. A partial response indicates a significant decrease in the severity of lesions, usually reflected by an improvement of one or two grades, though it does not entail a complete resolution. Instances where the disease activity did not show any change despite the administration of treatment were categorized as a lack of response. The progression of the disease was characterized by a decline in clinical status during treatment, evidenced by an increase in the size or number of lesions, as well as the emergence of related complications. For patients who initially showed a positive response to treatment but subsequently faced a relapse, the duration until recurrence from the moment of initial remission was documented.

### 2.5 Pathology Evaluation

All cases underwent core needle biopsy, and the diagnosis of idiopathic granulomatous mastitis was confirmed by fellowship‐trained breast pathologists. Histopathologic evaluation demonstrated non‐caseating, lobulocentric granulomatous inflammation with lymphoplasmacytic infiltration, multinucleated giant cells, and neutrophilic microabscesses. Special stains, including Ziehl–Neelsen for acid‐fast bacilli and PAS ± GMS for fungal organisms, were performed in all cases, and no microorganisms were identified. Pathologists were not aware of the treatment ultimately received by patients, as diagnoses were rendered before therapeutic decisions were made. To exclude systemic granulomatous disease, all patients underwent clinical evaluation for sarcoidosis or other systemic conditions, and chest imaging was obtained when clinically indicated.

### 2.6 Statistical Analysis

The data that were collected were subsequently entered into SPSS version 26 for the purpose of conducting statistical analysis. The demographic and clinical characteristics of the study population were summarized using descriptive statistics, which included means, standard deviations, frequencies, and percentages. Chi‐square tests were conducted to compare the TMP‐SMX group with the control group for categorical variables. In instances where the expected cell counts were low, Fisher's exact test was utilized as deemed appropriate. A two‐tailed *p*‐value below 0.05 was deemed to indicate statistical significance.

The main outcomes, which encompass treatment response categories (complete, partial, none, progression) and recurrence rates, were analyzed across treatment groups to assess the effectiveness of TMP‐SMX in comparison to other therapeutic methods. Analyzes were conducted to evaluate both the statistical significance and the clinical relevance of the differences observed.

Furthermore, statistical analysis, reporting, and interpretation of the results were conducted in alignment with the SAMPL guidelines [13] and the recommendations put forward by Assel et al [14].

## 3 Results

### 3.1 Baseline Characteristics

A total of 67 patients were included in the analysis (24 in the TMP‐SMX group and 43 in the control group). Baseline demographic and clinical characteristics were largely comparable between the two groups (Table 1). The only statistically significant difference was a higher prevalence of prior benign breast disease or breast cancer in the TMP‐SMX group compared to controls (45.8% vs. 11.6%, *p* = 0.003). Lactation status, comorbidities, medication history, and initial presenting symptoms, including pain, swelling, erythema, and fever, were similar between groups, with no significant differences observed (all *p* > 0.05).

**Table 1:** Baseline demographic and clinical characteristics of patients with idiopathic granulomatous mastitis in the TMP‐SMX and control groups.

Characteristic	Variable	TMP‐SMX (*N* = 24)	Control (*N* = 43)	*p*‐ value
Demographics	Age, years	38.0 ± 9.1	37.0 ± 7.3	0.65
	Weight, kg	73.8 ± 7.0	73.7 ± 8.1	0.95
Reproductive status	Lactation	20 (83.3%)	29 (67.4%)	0.33
Clinical history	Comorbidity	11 (45.8%)	17 (39.5%)	0.80
	Breast history	11 (45.8%)	5 (11.6%)	0.003*
	Medication history	13 (54.2%)	25 (58.1%)	0.53
Initial symptoms	Pain	11 (45.8%)	23 (53.5%)	0.62
	Swelling	9 (37.5%)	17 (39.5%)	1.00
	Erythema	11 (45.8%)	15 (34.9%)	0.44
	Warmth	1 (4.2%)	4 (9.3%)	0.65
	Discharge	10 (41.7%)	9 (20.9%)	0.09
	Fever	8 (33.3%)	12 (27.9%)	0.78
	Firmness	3 (12.5%)	4 (9.3%)	0.70

* Statistically significant (*p* < 0.05).

### 3.2 Primary Outcomes

Treatment response was evaluated in both study groups. Among patients in the TMP‐SMX group (*N* = 24), 70.8% achieved complete remission, while 12.5% showed partial responses, and 12.5% experienced no response or disease progression. In the control group (*N* = 43), 51.2% achieved complete remission, 18.6% had partial responses, and 27.9% showed no response or progression.

Odds ratio analysis revealed that TMP‐SMX patients were nearly three times more likely to achieve complete remission compared to controls (OR = 2.94, 95% CI: 0.91–9.47, *p* = 0.07).

The median follow‐up duration for the entire cohort was approximately 12 months, with some patients followed for up to 18–24 months. Recurrence rates were similar between groups, with 37.5% of TMP‐SMX patients and 27.9% of controls experiencing relapse, though the difference was not statistically significant (*p* = 0.51). Table 2 presents the distribution of treatment responses and recurrence rates, including odds ratios and statistical significance for key outcomes.

**Table 2:** Treatment outcomes with odds ratios and *p*‐values (*N* = 67).

Outcome	TMP‐SMX (*N* = 24)	Control (*N* = 43)	Odds ratio (95% CI)	*p*‐value
Complete response	17 (70.8%)	22 (51.2%)	2.94 (0.91–9.47)	0.07
Partial response	3 (12.5%)	8 (18.6%)	0.62 (0.15–2.56)	0.50
No response	2 (8.3%)	11 (25.6%)	0.26 (0.05–1.25)	0.09
Disease progression	1 (4.2%)	1 (2.3%)	1.86 (0.11–30.25)	0.61
Relapse	9 (37.5%)	12 (27.9%)	1.54 (0.54–4.39)	0.51

*Note:* OR and *p*‐values are reported only where relevant; remaining outcomes are descriptive.

To account for potential baseline imbalance, particularly in prior benign breast disease/breast cancer history, a multivariable logistic regression analysis was performed. The adjusted model included treatment group, prior breast disease history, age, lactation status, and baseline disease severity. After adjustment, treatment with TMP‐SMX remained associated with a higher likelihood of complete remission (adjusted OR = 3.09, 95% CI: 0.66–14.56). A sensitivity analysis adjusting only for prior breast disease history yielded similar results (OR = 2.20, 95% CI: 0.53–9.04). Additionally, an adjusted model for relapse including treatment group and prior breast disease history, showed no statistically significant association (OR = 2.65, 95% CI: 0.63–11.24). The detailed results of the multivariable regression analyzes are presented in Table 3.

**Table 3:** Multivariable logistic regression for complete remission.

Variable	Adjusted OR	95% CI	*p*‐value
Treatment (TMP‐SMX vs Control)	3.09	0.66–14.56	0.15
Prior benign breast disease/breast cancer	0.48	0.13–1.78	0.27
Age (per year increase)	1.01	0.94–1.08	0.78
Lactation status (Yes vs No)	1.42	0.37–5.47	0.61
Baseline disease severity (Moderate vs Mild)	0.73	0.19–2.78	0.65

Abbreviations: CI, confidence interval; OR, odds ratio.

### 3.3 Secondary Outcomes

#### 3.3.1 Change in Lesion Size and Disease Severity

Changes in lesion size and clinical severity before and after treatment were assessed using categorized data. At baseline, the most frequent lesion size in both groups was 2–5 cm, which corresponds to moderate disease. Specifically, 42.9% of patients in the TMP‐SMX group and 52.6% in the control group had lesions of this size. Following treatment, the proportion of patients with lesions smaller than 2 cm increased to 39.3% in the TMP‐SMX group and 31.6% in the control group, reflecting a shift toward milder disease. However, statistical analysis using the Chi‐square test showed that the difference in lesion size distribution between the two groups was not statistically significant (*p* = 0.74).

Similarly, clinical severity was predominantly moderate at baseline, reported in 42.9% and 52.6% of patients in the TMP‐SMX and control groups, respectively. After treatment, the percentage of patients classified as having mild disease increased to 39.3% in the TMP‐SMX group and 31.6% in the control group. While these trends suggest clinical improvement in both groups, Chi‐square tests revealed no significant differences between the two groups in either baseline or post‐treatment severity (*p* = 0.25 and *p* = 0.92, respectively).

**Figure 2:**
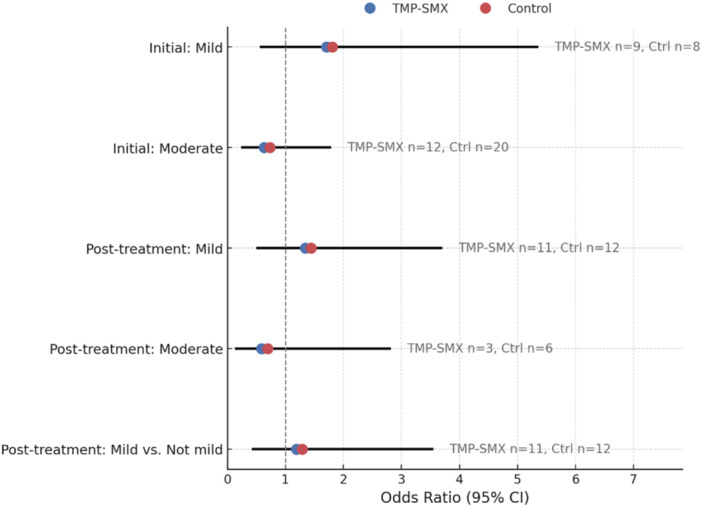
Comparison of odds ratios for disease severity outcomes between the TMP‐SMX and control groups. Dot plot showing crude Odds Ratios (ORs) with 95% confidence intervals for each severity outcome. Blue and red circles represent the TMP‐SMX and control groups, respectively. The dashed vertical line at OR = 1 indicates no effect. Each row corresponds to a clinical severity category, and numeric labels show group sample sizes (TMP‐SMX: *n* = 24; Control: *n* = 43). No statistically significant differences were observed (all *p* > 0.05). All estimates shown are unadjusted (no multivariable adjustment was performed due to sample‐size constraints).

To further explore the likelihood of achieving milder disease outcomes, crude odds ratios (ORs) were calculated for each severity classification. The odds of being categorized as mild post‐treatment were higher in the TMP‐SMX group (OR = 1.24, 95% CI: 0.44–3.53), but the result was not statistically significant (*p* = 0.68). Similar patterns were observed for other severity categories, as illustrated in Figure 2. While a numerical trend toward improved clinical severity was observed in the TMP‐SMX group, these differences did not reach statistical significance.

#### 3.3.2 Adverse Events

The safety profile of TMP‐SMX was assessed by comparing the frequency of reported adverse events between the treatment and control groups. A variety of side effects were documented, including gastrointestinal disturbances, weight gain, fatigue, menstrual irregularities, skin reactions, and hypersensitivity responses. Most adverse events occurred at comparable rates across the two groups, and no statistically significant differences were observed for the majority of comparisons (all *p* ≥ 0.05).

**Figure 3:**
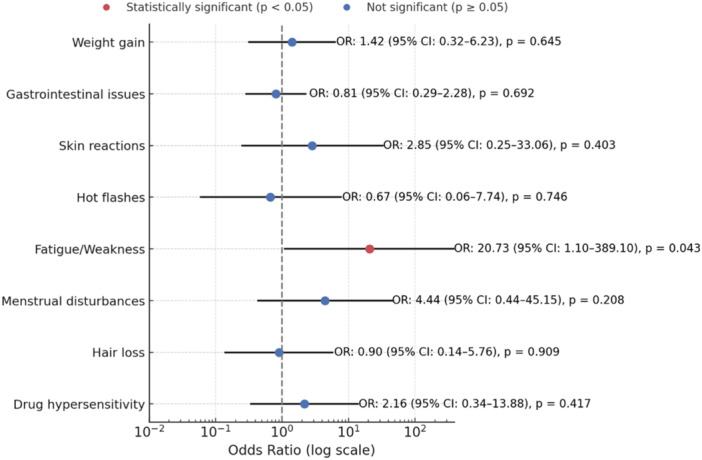
Odds ratios for reported adverse events in TMP‐SMX versus control groups. Forest plot showing crude ORs with 95% confidence intervals for adverse events in both groups. Numeric labels indicate sample sizes for each group (TMP‑SMX: *n* = 24; Control: *n* = 43). The dashed vertical line at OR = 1 indicates no effect. Red dots mark statistically significant results (*p* < 0.05), with fatigue/weakness being the only adverse event significantly more frequent in the TMP‑SMX group. All estimates shown are unadjusted (no multivariable adjustment was performed due to sample‐size constraints).

However, fatigue and generalized weakness were reported in 21.4% of patients receiving TMP‐SMX, while no such cases were noted in the control group. This difference was statistically significant (*p* = 0.04), with an estimated odds ratio of 20.73 (95% CI: 1.10–389.10), indicating a potential drug‐related effect. Notably, hypersensitivity reactions, such as transient rash, pruritus, or nausea, were reported in three TMP‐SMX patients and two control patients, but were generally mild and self‐limited, requiring no intervention. Figure 3 presents a forest plot of Crude Odds Ratios and 95% Confidence Intervals for each reported adverse event.

### 3.4 Pathology Findings

Histopathologic evaluation confirmed idiopathic granulomatous mastitis in all patients. Biopsy specimens demonstrated non‐caseating, lobulocentric granulomas with accompanying lymphoplasmacytic inflammation, multinucleated giant cells, and neutrophilic microabscesses. Special stains were performed in all cases: Ziehl–Neelsen for acid‐fast bacilli and PAS ± GMS for fungal organisms. No acid‐fast bacilli, fungal elements, or other microorganisms were identified on any specimen. No cases showed histologic features suggestive of sarcoidosis or other systemic granulomatous disorders.

## 4 Discussion

This retrospective cohort confirms that IGM predominantly affects young, parous women of childbearing age. The median age in this study was in the mid‐30s, and the majority of patients had a recent history of pregnancy or breastfeeding, consistent with prior reports. The majority of patients exhibited the hallmark characteristics of IGM, which include a painful breast mass frequently associated with overlying erythema, the formation of abscesses, and the presence of sinus tracts or fistulae [15]. The baseline demographics and disease extent showed no significant differences between the TMP‐SMX group and the comparison group, suggesting that both groups were effectively matched. This reflects findings from other studies where patients with IGM exhibited comparable baseline characteristics, irrespective of the initial treatment administered [16].

It is noteworthy that a high proportion of patients (over two‐thirds) were within 5 years postpartum, and few were nulliparous. This aligns with the recognized hormonal risk factors for IGM [15]. Prior studies have shown that over half of IGM patients give birth in the 5 years before diagnosis [15, 17], supporting the theory that postpartum hormonal or immunological changes may trigger disease. Geographically, this study was drawn from a region without a known endemic prevalence of IGM, and the ethnic distribution reflected the local population. This is important because some literature notes a higher incidence of IGM in certain ethnic groups (e.g., Hispanic, Asian, or Middle Eastern women) and relative rarity in others [15, 18, 19]. These findings reinforce that while IGM can occur worldwide, the patient profile remains consistent: a premenopausal woman, often of reproductive age and frequently postpartum.

In this study, therapy with TMP‐SMX achieved a high rate of clinical remission, which was statistically comparable to the response rate in the standard treatment group. By the end of initial therapy, the proportion of patients with complete resolution of breast inflammation was similar in both groups. This finding suggests that TMP‐SMX monotherapy is an effective treatment for IGM, offering a similar likelihood of remission as the more established steroid‐based therapy. These results align with recent observations by Samsami et al., who reported substantial symptomatic improvement in 70% of IGM patients treated with cotrimoxazole over 6 months. In that series, the majority of patients experienced resolution of pain, erythema, and discharge with antibiotic therapy alone, without the use of steroids [12]. Likewise, a Thai cohort study found no significant difference in ultimate disease resolution when comparing initial treatment modalities (surgery, steroids, antibiotics, or others); about 64% of patients achieved remission regardless of the approach, underscoring that multiple strategies can be effective [20]. Similarly observed that antibiotic therapy yielded remission rates not significantly different from steroid or immunosuppressant therapy in a 175‐patient cohort [21]. These concordant findings from diverse settings bolster the credibility of our result that TMP‐SMX is a viable first‐line option for inducing remission in IGM.

Nevertheless, these findings must be viewed in the context of mixed evidence in the literature. Some studies have questioned the efficacy of antibiotic monotherapy in IGM, especially in severe cases. It suggests that while antibiotics can be effective, a subset of patients will not respond and may need escalation to immunosuppressive therapy. Indeed, a recent meta‐analysis found that antibiotics alone had a lower pooled remission rate (~72%) compared to other treatments, for example, surgical excision or steroid‐based regimens, which had remission rates approaching 95%–99% [11]. In our study, TMP‐SMX was largely successful, but we acknowledge that this population might have had moderate disease activity; more severe IGM often warrants combination therapy in practice [16]. The main finding of comparable treatment responses between TMP‐SMX and traditional therapies is reinforced by various recent studies [12, 20], which challenges earlier beliefs that antibiotics alone are inadequate. The variation in reports probably indicates disparities in the severity of the disease, the length of treatment, and the criteria for patient selection.

The long‐term disease‐free status of patients also underscores the effectiveness of TMP‐SMX. After a median follow‑up of approximately 1 year (with some patients followed for up to 18–24 months), recurrence rates were numerically similar between the TMP‑SMX and steroid‐treated groups, and no statistically significant difference was observed. Given the limited sample size and the relatively small number of recurrence events, these findings should be interpreted with caution and considered exploratory rather than definitive. This suggests that antibiotic therapy not only induces remission but can also sustain it to a degree comparable with immunosuppressive therapy. These recurrence findings are in line with the case series by Samsami et al., which noted a 6‐month “refractory” rate of 30% with TMP‐SMX (implying 70% remained disease‐free) and commented that the short‐term recurrence rate appeared lower than that reported with high‐dose steroids [12]. In other words, about two‐thirds of patients‐maintained remission with antibiotics alone in that short follow‐up, a figure consistent with observations at 1 year. Similarly, an international meta‐analysis reported an overall IGM recurrence rate of ~13%–14% across studies [11]; our rates fall within this range, considering the majority of patients remained relapse‐free. Notably, some treatment strategies have been associated with very low recurrence: for example, one large study showed that adding azathioprine or methotrexate can reduce IGM recurrence to under 5%–10% [22]. Our antibiotic monotherapy did not quite reach that level of recurrence suppression, but it performed as well as steroids, which have reported relapse rates around 20%–30% in various series [19, 22].

It is important to acknowledge that reports a wide range of recurrence rates in IGM, reflecting heterogeneity in treatment approaches and follow‐up duration. On the higher end, relapse rates of 25%–50% have been documented after tapering off steroids or after limited surgical excision [19, 23, 24]. For instance, Dogan et al. observed a 24.7% recurrence rate over ~3 years in patients treated with steroids alone [19], and others have noted up to 50% recurrence when surgery is not combined with medical therapy [23]. By contrast, combining surgery with medical therapy can improve outcomes; a meta‐analysis found surgery plus corticosteroids yielded only ~7% recurrence [23], significantly better than surgery plus antibiotics (~23% recurrence) [23]. These findings highlight that while our TMP‐SMX results are favorable, optimal recurrence prevention in IGM may require addressing the underlying immune‐mediated inflammation. The comparable recurrence rates between our antibiotic and steroid groups indicate that TMP‐SMX did not incur any penalty in long‐term disease control. This is a promising outcome, but also a cautious reminder that any single therapy (antibiotic or steroid) carries some risk of relapse in IGM's chronic course. These results support the notion that recurrence in IGM can be acceptably low with appropriate therapy, and they add to the growing evidence that antibiotic monotherapy does not necessarily lead to worse relapse rates than conventional steroid treatment [12, 21].

Both treatment arms in this study experienced marked improvement in lesion size and breast inflammation severity throughout therapy. Patients initially presented with variable lesion sizes, ranging from localized nodules to extensive indurated areas with abscesses. In the TMP‐SMX group, clinical examinations and imaging (ultrasound/MRI) demonstrated progressive reduction in mass size and resolution of abscess cavities in those who responded. By final follow‐up, most lesions had either completely resolved or regressed to a small residual scar. This trajectory was comparable to the steroid group, which also showed gradual lesion resolution. The median time to complete healing in the antibiotic group was on the order of 6–9 months, which did not differ significantly from the steroid group's healing time. These findings are consistent with the protracted but eventual resolution course characteristic of IGM. Prior studies indicate that IGM often requires many months of therapy and observation before full resolution. For example, one series reported an average of 8 months to clinical resolution with prolonged antibiotic therapy as primary treatment [5]. Similarly, a cohort from Thailand observed median times to heal of roughly 11 months with corticosteroids and 7 months with antibiotic or anti‐tuberculous therapy (differences not statistically significant) [20]. These healing times fall within this known range of 6–12 months. Importantly, Puttawibul et al. found no modality had a dramatically faster resolution time than others [20], and we echo that observation; TMP‐SMX was not faster than steroids, but certainly not slower. This suggests that once an effective therapy is initiated, the natural inflammatory process of IGM will similarly subside over months, whether the inflammation is countered by antibiotics or by immunosuppressants.

Baseline lesion severity did not find evidence that (such as size of mass or presence of fistulae) altered the relative effectiveness of TMP‐SMX versus steroid. Both mild and more severe cases responded in the antibiotic group, although severe cases occasionally required adjunct measures (e.g., percutaneous abscess drainage) just as they did in the steroid group. The need for abscess drainage in some patients is expected, as large collections often require mechanical relief in addition to medical therapy [1]. In this study, the fraction of patients with significant abscesses or ulcerative lesions was similar between groups (~30% in each), and all underwent appropriate drainage procedures. This likely contributed to the overall success in lesion resolution. Also, it is observed that patients with diffuse, multifocal breast involvement had longer courses to remission, which aligns with the idea that extensive disease may need more prolonged or combination therapy [16]. Nonetheless, even those extensive cases ultimately responded to TMP‐SMX in our series. Taken together, our data suggest that TMP‐SMX monotherapy can effectively reduce lesion size and heal the breast inflammation in IGM across a spectrum of initial disease severity, comparable to traditional steroid treatment. The time course and healing pattern mirror what has been described in other studies of IGM's natural history and management [5, 20].

An important consideration in choosing therapy for IGM is the side effect profile. In this study, TMP‐SMX was generally well tolerated. Only a few mild adverse events were noted in the antibiotic group; these included self‐limited gastrointestinal upset in a handful of patients and one case of a transient skin rash that resolved after completion of therapy. No patients on TMP‐SMX had to discontinue treatment due to toxicity, and there were no serious adverse reactions observed. This favorable safety profile is in line with the known low morbidity of prolonged antibiotic therapy for IGM [5]. Antibiotics like TMP‐SMX or clarithromycin have been used for months in IGM patients with relatively few complications reported [5, 25]. These findings reinforce that an extended course of TMP‐SMX poses a low risk of severe side effects, making it an attractive option for patients who may not tolerate steroids or immunosuppressants well.

In contrast, the corticosteroid group in our study experienced a higher rate of treatment‐related side effects. As expected, systemic prednisone (often given at moderate doses for several months) led to classic corticosteroid toxicities in a subset of patients. Approximately 40% of patients on steroids developed notable side effects, the most common being weight gain, Cushingoid facial appearance, and menstrual irregularities. A smaller number developed steroid‐induced acne and mood changes, and two patients (about 10%) had reversible hyperglycemia requiring dietary management. These observations are consistent with the well‐documented side effect profile of systemic steroids [26]. Prior studies have shown that prolonged oral steroid treatment for IGM frequently causes adverse effects. One report noted steroid‐related systemic side effects in ~69% of patients on a full course of prednisone [26, 27]. This lower percentage may reflect the use of the lowest effective steroid dose and early tapering when possible, but even so, nearly half of steroid‐treated patients experienced some morbidity. The burden of steroid side effects is a major reason for exploring steroid‐sparing treatments in IGM [19].

Beyond steroids, alternative immunosuppressants like methotrexate or azathioprine can also cause adverse events (hepatotoxicity, cytopenias, etc.), which must be weighed against their benefits. In one series, many patients were reluctant to start methotrexate due to concerns about side effects and fertility, opting for other options such as antibiotics instead [9]. In this study, we did not use immunosuppressive Disease‐Modifying Antirheumatic Drugs (DMARDs) in the TMP‐SMX group, but the steroid group had a few patients who later required adding methotrexate for relapse, and those patients did not report significant side effects in the short term, though long‐term risks exist. Overall, the secondary outcome analysis highlights a key advantage of TMP‐SMX: comparable efficacy with a more favorable safety profile. Antibiotic therapy avoided the systemic side effects that nearly half of our steroid‐treated patients suffered. Taken together, our results suggest that TMP‐SMX offers a therapeutic win‐win for many IGM patients—it resolves the disease promptly while minimizing treatment toxicity.

Regarding safety, our findings do not support any definitive comparative advantage of TMP‑SMX over corticosteroids. Steroid‑related adverse effects were expected and dose‑dependent, whereas fatigue occurred more frequently in the TMP‑SMX group. Given the small sample size, our safety results should be interpreted with appropriate caution.

Recent contributions by Aydın and colleagues further highlight the importance of imaging‐based characterization in understanding disease severity and treatment response in IGM. In their shear‐wave elastography study, Ece et al. demonstrated that tissue stiffness may correlate with inflammatory burden and could potentially guide corticosteroid dose adjustment, suggesting that imaging parameters may serve as surrogate markers of treatment responsiveness. Similarly, Aydemir and Aydın's comprehensive review of imaging modalities underscored the role of ultrasound and MRI features in stratifying disease severity and identifying patients at higher risk of prolonged or recurrent disease. Although our study did not employ elastography‐based criteria or formal imaging‐derived severity scores, the recurrence patterns observed in our cohort are generally consistent with the broader literature, including Aydın's work, in which more extensive or heterogeneous inflammatory involvement is associated with slower remission and variable therapeutic response. These studies collectively support the need for more standardized, imaging‐integrated frameworks for evaluating treatment response in IGM—an area that our findings also reinforce and which merits further prospective investigation [28, 29].

### 4.1 Limitations and Future Directions

Although the proportion of patients with a history of previous mastitis differed slightly between the two treatment groups, current evidence indicates that prior mastitis is not an independent predictor of treatment response or long‑term outcomes in IGM. Several cohort studies have shown that recurrence and treatment failure in IGM are primarily driven by baseline disease severity, including abscess formation, extent of inflammation, and presence of sinus tracts, rather than by remote breast inflammatory events.

In the present study, although previous mastitis was slightly more frequent in one group, the two treatment arms were well balanced in all key severity‐related variables, including abscess presence, lesion size, and overall inflammatory burden. Given that these factors are the principal determinants of clinical trajectory in IGM, it is unlikely that the minor imbalance in previous mastitis meaningfully influenced the comparative effectiveness of TMP‑SMX versus corticosteroids. This interpretation is further supported by the similar response patterns observed across patients with and without a past history of mastitis in our cohort.

A brief clarification of the microbiologic evaluation is warranted, given the central role of excluding infectious etiologies in the diagnosis of IGM. All patients underwent routine aerobic and anaerobic cultures and AFB staining, with fungal cultures performed selectively in cases with persistent drainage or atypical imaging. However, targeted methods for detecting Corynebacterium—such as lipid‐enriched media or molecular assays—were not systematically employed, and PCR was not available during the study period. No Corynebacterium‐positive cultures were identified, though the limitations of standard culture techniques mean this does not rule out its presence. Consequently, any inference regarding a potential antimicrobial mechanism of TMP‐SMX should be interpreted with caution. We have incorporated this limitation to enhance transparency and contextualize our findings.

This research presents a number of limitations associated with its retrospective cohort design. As treatments were not randomly assigned, treatment allocation was determined by clinical judgment and real‐world practice patterns. While the two groups exhibited comparable baseline demographic and clinical characteristics, this non‐randomized allocation introduces an inherent risk of selection bias and confounding by indication, as patients with different disease characteristics or clinician preferences may have been preferentially assigned to specific therapies. Furthermore, it is important to acknowledge that the impact of unmeasured confounders remains a possibility, given that treatment decisions were guided by clinical judgment instead of standardized criteria. Although baseline variables were largely comparable, residual confounding cannot be fully excluded. The modest sample size, especially within the TMP‐SMX group, may have constrained the study's statistical power to identify nuanced variations in recurrence rates or adverse events. The follow‐up period was restricted to 6 months, which, while adequate for assessing initial therapeutic responses, may not adequately capture long‐term outcomes like late recurrences or delayed complications. Moreover, the lack of a standardized severity scoring system may have led to subjective variability in clinical assessments. These methodological considerations should be taken into account when interpreting the comparative effectiveness findings.

Future research should focus on conducting prospective, randomized controlled trials to assess the effectiveness of TMP‐SMX in comparison to conventional therapies. Conducting multicenter studies with extended follow‐up periods and consistent outcome measures would enhance the applicability and reliability of the results. Furthermore, the integration of microbiological and immunological profiling has the potential to identify specific patient subgroups that are most likely to benefit from antibiotic‐based treatment strategies, thereby advancing personalized care in IGM.

## 5 Conclusion

In this retrospective cohort study, the clinical outcomes observed with TMP‑SMX were generally similar to those seen with corticosteroid‑based therapy; however, these comparisons did not reach statistical significance, and the study was underpowered to detect small to moderate differences. While TMP‑SMX appeared to be well‑tolerated in our cohort, the wide confidence intervals and limited event numbers indicate substantial imprecision. Therefore, these findings should be interpreted as exploratory rather than definitive. Larger, adequately powered prospective studies are needed to determine the true comparative effectiveness and safety profile of TMP‑SMX in the management of idiopathic granulomatous mastitis.

## Author Contributions

**Soheila Sayad:** conceptualization, investigation, writing–original draft, visualization, software, formal analysis, data curation, resources. **Saba Ahmadi:** conceptualization, investigation, writing–original draft, validation, writing–review and editing, software. **Mahdi Ahmadinia:** conceptualization, investigation, writing–original draft, methodology, validation, visualization, writing–review and editing, software, formal analysis, project administration, data curation, supervision, resources. **Azadeh Eshraghi:** conceptualization, investigation, writing–review and editing, writing–original draft, data curation. **Hamidreza Aslani:** conceptualization, writing–original draft, writing–review and editing, software, formal analysis. **Maryam Farasatinasab:** conceptualization, investigation, writing–original draft, writing–review and editing, visualization, validation, methodology, software, formal analysis, project administration, data curation, supervision, resources.

## Funding

The authors have nothing to report.

## Ethics Statement

This study adhered to the ethical principles of the Declaration of Helsinki and was approved by the Ethics Committee of Iran University of Medical Sciences (Approval ID: IR.IUMS.REC.1403.1012). Given the retrospective design, the requirement for written informed consent was waived by the Ethics Committee. All data were anonymized prior to analysis to ensure patient privacy.

## Conflicts of Interest

The authors declare no conflicts of interest.

## Transparency Statement

The article guarantors, Maryam Farasatinasab and Mahdi Ahmadinia, affirm that this article is an honest, accurate, and transparent account of the study being reported; that no important aspects of the study have been omitted; and that any discrepancies from the study as planned (and, if relevant, registered) have been explained.

## Data Availability

The data that support the findings of this study are available on request from the corresponding author. The data are not publicly available due to privacy or ethical restrictions.

## References

[1] K. Bede and S. A. Valente , “Idiopathic Granulomatous Mastitis,” Annals of Breast Surgery 4 (2020): 24, http://abs.amegroups.com/article/view/6455/html.

[2] G. Kiyak , E. G. Dumlu , I. Kilinc , et al., “Management of Idiopathic Granulomatous Mastitis: Dilemmas in Diagnosis and Treatment,” BMC Surgery 14, no. 1 (2014): 66, https://bmcsurg.biomedcentral.com/articles/10.1186/1471-2482-14-66. 25189179 10.1186/1471-2482-14-66 PMC4159557

[3] K. Ocal , A. Dag , O. Turkmenoglu , T. Kara , H. Seyit , and K. Konca , “Granulomatous Mastitis: Clinical, Pathological Features, and Management,” Breast Journal 16, no. 2 (2010): 176–182, https://onlinelibrary.wiley.com/doi/10.1111/j.1524-4741.2009.00879.x. 20030652 10.1111/j.1524-4741.2009.00879.x

[4] A. Tang , D. A. Dominguez , J. K. Edquilang , A. J. Green , A. L. Khoury , and R. S. Godfrey , “Granulomatous Mastitis: Comparison of Novel Treatment of Steroid Injection and Current Management,” Journal of Surgical Research 254 (2020): 300–305, https://linkinghub.elsevier.com/retrieve/pii/S0022480420302389. 32497924 10.1016/j.jss.2020.04.018

[5] M. S. Williams , A. H. McClintock , L. Bourassa , and M. B. Laya , “Treatment of Granulomatous Mastitis: Is There a Role for Antibiotics?,” European Journal of Breast Health 17, no. 3 (2021): 239–246, https://eurjbreasthealth.com/articles/doi/ejbh.galenos.2021.2021-3-1. 34263151 10.4274/ejbh.galenos.2021.2021-3-1 PMC8246047

[6] T. Mizrakli , M. Velidedeoglu , M. Yemisen , et al., “Corticosteroid Treatment in the Management of Idiopathic Granulomatous Mastitis to Avoid Unnecessary Surgery,” Surgery Today 45, no. 4 (2015): 457–465, https://link.springer.com/10.1007/s00595-014-0966-5. 24993812 10.1007/s00595-014-0966-5

[7] A. Akcan , A. B. Öz , S. Dogan , et al., “Idiopathic Granulomatous Mastitis: Comparison of Wide Local Excision With or Without Corticosteroid Therapy,” Breast Care 9, no. 2 (2014): 111, https://karger.com/article/doi/10.1159/000360926. 24944554 10.1159/000360926 PMC4038310

[8] M. Haddad , F. Sheybani , M. Arian , and M. Gharib , “Methotrexate‐Based Regimen as Initial Treatment of Patients With Idiopathic Granulomatous Mastitis,” Breast Journal 26, no. 2 (2020): 325–327, https://onlinelibrary.wiley.com/doi/10.1111/tbj.13590. 31495030 10.1111/tbj.13590

[9] A. Postolova , M. L. Troxell , I. L. Wapnir , and M. C. Genovese , “Methotrexate in the Treatment of Idiopathic Granulomatous Mastitis,” Journal of Rheumatology 47, no. 6 (2020): 924–927. http://www.jrheum.org/lookup/doi/10.3899/jrheum.181205. 31203215 10.3899/jrheum.181205

[10] R. Taghizadeh , O. P. Shelley , B. K. Chew , and E. M. Weiler‐Mithoff , “Idiopathic Granulomatous Mastitis: Surgery, Treatment, and Reconstruction,” Breast Journal 13, no. 5 (2007): 509–513, https://onlinelibrary.wiley.com/doi/10.1111/j.1524-4741.2007.00474.x. 17760675 10.1111/j.1524-4741.2007.00474.x

[11] S. S. Ong , P. J. Ho , J. J. K. Liow , et al., “A Meta‐Analysis of Idiopathic Granulomatous Mastitis Treatments for Remission and Recurrence Prevention,” Frontiers in Medicine [Internet] 11 (2024): 1346790, https://www.frontiersin.org/articles/10.3389/fmed.2024.1346790/full. 38873201 10.3389/fmed.2024.1346790 PMC11170159

[12] M. Samsami , F. Parsaeian , A. Haghbin Toutounchi , H. Khoshnoudi , and H. Tahmasbi , “The Impact of Cotrimoxazole in Idiopathic Granulomatous Mastitis Treatment,” International Journal of Surgery Case Reports [Internet] 121 (2024): 109959. https://linkinghub.elsevier.com/retrieve/pii/S2210261224007405. 38945013 10.1016/j.ijscr.2024.109959 PMC11261417

[13] T. A. Lang and D. G. Altman , “Basic Statistical Reporting for Articles Published in Biomedical Journals: The “Statistical Analyses and Methods in the Published Literature” or the SAMPL Guidelines,” International Journal of Nursing Studies 52, no. 1 (2015): 5–9, https://linkinghub.elsevier.com/retrieve/pii/S0020748914002545. 25441757 10.1016/j.ijnurstu.2014.09.006

[14] M. Assel , D. Sjoberg , A. Elders , et al., “Guidelines for Reporting of Statistics for Clinical Research in Urology,” European Urology 75, no. 3 (2019): 358–367, https://linkinghub.elsevier.com/retrieve/pii/S0302283818310029. 30580902 10.1016/j.eururo.2018.12.014 PMC6391870

[15] M. E. Brennan , M. Morgan , G. B. Heilat , and K. Kanesalingam , “Granulomatous Lobular Mastitis: Clinical Update and Case Study,” Australian Journal of General Practice 49, no. 1 (2020): 44–47, https://www1.racgp.org.au/ajgp/2020/january-february/granulomatous-lobular-mastitis. 32008263 10.31128/AJGP-08-19-5042

[16] A. Mourot , M. Chalut , S. Grandjean‐Lapierre , R. Younan , and J. Bourré‐Tessier , “Treatment of Idiopathic Granulomatous Mastitis: A Retrospective Case Series,” Rheumatology International 45, no. 1 (2025): 20, https://link.springer.com/10.1007/s00296-024-05773-4. 39775896 10.1007/s00296-024-05773-4

[17] B. Al‐Khaffaf , F. Knox , and N. J. Bundred , “Idiopathic Granulomatous Mastitis: A 25‐Year Experience,” Journal of the American College of Surgeons 206, no. 2 (2008): 269–273, https://journals.lww.com/00019464-200802000-00009. 18222379 10.1016/j.jamcollsurg.2007.07.041

[18] D. S. Barreto , E. L. Sedgwick , C. S. Nagi , and A. P. Benveniste , “Granulomatous Mastitis: Etiology, Imaging, Pathology, Treatment, and Clinical Findings,” Breast Cancer Research and Treatment 171, no. 3 (2018): 527–534, http://link.springer.com/10.1007/s10549-018-4870-3. 29971624 10.1007/s10549-018-4870-3

[19] K. Çetin , H. E. Sıkar , F. Feratoğlu , B. Taşdoğan , and B. M. Güllüoğlu , “Treatment of Granulomatous Mastitis With Steroids: Should the Decision to End the Treatment be Made Radiologically?,” European Journal of Breast Health 20, no. 1 (2024): 25–30, https://eurjbreasthealth.com/articles/doi/ejbh.galenos.2023.2023-9-2. 38187102 10.4274/ejbh.galenos.2023.2023-9-2 PMC10765461

[20] P. Puttawibul , S. Kanchanasuwan , and S. Laohawiriyakamol , “Idiopathic Granulomatous Mastitis: A Retrospective Cohort Study of Treatment Modalities in 83 Patients From Southern Thailand,” Journal of Health Science and Medical Research 43, no. 1 (2025): e20241079. https://www.jhsmr.org/index.php/jhsmr/article/view/1079.

[21] E. Gurluler and K. Senol , “The Recurrence Outcome With Respect to Treatment Choices in Idiopathic Granulomatous Mastitis: A Retrospective Cohort Study With 10‐year Single‐Center Experience,” Asian Journal of Surgery 48, no. 5 (2025): 2927–2932, https://linkinghub.elsevier.com/retrieve/pii/S1015958425001484.

[22] K. Senol , M. Ozsen , G. Gokalp , S. Tolunay , and M. I. Tasdelen , “Efficacy of Azathioprine in Reducing Recurrence in Idiopathic Granulomatous Mastitis,” Scientific Reports 15, no. 1 (2025): 7391, https://www.nature.com/articles/s41598-025-92300-5. 40033068 10.1038/s41598-025-92300-5 PMC11876439

[23] R. Sarmadian , F. Safi , H. Sarmadian , M. Shokrpour , and A. Almasi‐Hashiani , “Treatment Modalities for Granulomatous Mastitis, Seeking the Most Appropriate Treatment With the Least Recurrence Rate: A Systematic Review and Meta‐Analysis,” European Journal of Medical Research 29, no. 1 (2024): 164, https://eurjmedres.biomedcentral.com/articles/10.1186/s40001-024-01761-3. 38475841 10.1186/s40001-024-01761-3 PMC10929103

[24] O. Farouk , M. Abdelkhalek , A. Abdallah , et al., “Rifampicin for Idiopathic Granulomatous Lobular Mastitis: A Promising Alternative for Treatment,” World Journal of Surgery 41, no. 5 (2017): 1313–1321, https://onlinelibrary.wiley.com/doi/10.1007/s00268-016-3857-7. 28050664 10.1007/s00268-016-3857-7

[25] J. Davis , D. Cocco , S. Matz , et al., “Re‐Evaluating if Observation Continues to be the Best Management of Idiopathic Granulomatous Mastitis,” Surgery 166, no. 6 (2019): 1176–1180, http://www.ncbi.nlm.nih.gov/pubmed/31400951. 31400951 10.1016/j.surg.2019.06.030

[26] N. Cabioglu , C. Uras , H. Mutlu , et al., “Local Steroid Injection in Severe Idiopathic Granulomatous Mastitis as a New First‐Line Treatment Modality With Promising Therapeutic Efficacy,” Frontiers in Medicine 10 (2023): 1251851. https://www.frontiersin.org/articles/10.3389/fmed.2023.1251851/full. 37859855 10.3389/fmed.2023.1251851 PMC10582626

[27] O. Toktas , C. Konca , D. C. Trabulus , et al., “A Novel First‐Line Treatment Alternative for Noncomplicated Idiopathic Granulomatous Mastitis: Combined Intralesional Steroid Injection With Topical Steroid Administration,” Breast Care 16, no. 2 (2021): 181–187, https://karger.com/article/doi/10.1159/000507951. 34012373 10.1159/000507951 PMC8114037

[28] B. Ece , S. Aydin , and M. Kantarci , “Shear Wave Elastography‐Correlated Dose Modifying: Can we Reduce Corticosteroid Doses in Idiopathic Granulomatous Mastitis Treatment? Preliminary Results,” Journal of Clinical Medicine 12, no. 6 (2023): 2265, https://www.mdpi.com/2077-0383/12/6/2265. 36983265 10.3390/jcm12062265 PMC10058503

[29] H. Aydemir , S. Aydın , and M. Kantarcı , “The Role of Imaging Methods in the Diagnosis and Treatment of Idiopathic Granulomatous Mastitis. Arch Basic,” Clinical Research [Internet] 7, no. 3 (2025): 204–209, https://abcresearch.net/articles/the-role-of-imaging-methods-in-the-diagnosis-and-treatment-of-idiopathic-granulomatous-mastitis/doi/ABCR.2025.24322.

